# A flow cytometry based method for studying embryogenesis and immune reactivity to embryogenic stages in filarial parasites

**DOI:** 10.1186/1475-2883-4-11

**Published:** 2005-11-07

**Authors:** Bikash Ranjan Sahu, Alok Das Mohapatra, Arindam Majumder, Pradip K Das, Balachandran Ravindran

**Affiliations:** 1 Division of Immunology, Regional Medical Research Centre, Indian Council of Medical Research, Chandrasekarpur, Bhubaneswar, 751023, India; 2 Division of Microbiology, National Institute of Cholera and Enteric Diseases, Kolkata, 70000, India

## Abstract

**Background:**

In the absence of intermediate animal hosts, the process of embryogenesis leading to fecundity of adult female filarial worms is very critical for persistence of these obligate parasites in human communities. Embryogenesis in adult female filarial parasites involves fertilization of eggs or oocytes by sperms and their subsequent development into motile microfilariae inside the uterine cavity of worms. Development of assays for monitoring embryogenesis in adult female worms is a critical requirement in filariasis research – filarial worms are known to harbour endosymbionts such as *Wolbachia *which play a significant role in fecundity. Tetracycline or doxycycline treatment of the infected hosts effectively eliminates the endosymbionts resulting in inhibition of embryogenesis in female worms. Currently, inhibition of embryogenesis in adult filarial worms can be monitored only by microscopic examination of *in vitro *harvested intrauterine stages.

**Methods:**

Adult female filarial worms of bovine filarial parasites, *Setaria digitata *were collected from the peritoneum of infected animals and intrauterine stages were harvested in culture medium and were analyzed for forward and side scatter by flowcytometry using a BD FACS Calibur. Different populations were gated, sorted and identified by phase microscopy. Binding of biotinylated lectins to intra uterine stages was monitored using FITC labeled Avidin and monitored by flow cytometry of gated populations. Similarly, binding of antibodies in human filarial sera to intrauterine stages was monitored using FITC labeled anti-human immunoglobulins.

**Results:**

The forward and side scatter for intrauterine stages delineated 3 distinct populations labeled as R1, R2 and R3. The three populations were sorted and identified to be a) fully stretched microfilariae, b) early and c) late developmental stages of eggs respectively. Lectins such as Wheat Germ agglutinin or Concanavalin-A were found to bind strongly to egg stages and less prominently to intra-uterine microfilariae. Similarly the binding of antibodies in filarial sera to the three intra-uterine stages could also be precisely quantified.

**Conclusion:**

The manuscript reports a novel flow cytometry based method to monitor progression of embryogenesis in adult filarial worms. Apart from relative quantification of different intra uterine developmental stages, the assay allows quantitative binding of lectins and antibodies to each of the intrauterine stages. It may now be possible to quantify levels of antibodies in infected and immune hosts to monitor anti-fecundity immunity in filariasis – the assay can thus be used as a powerful tool for drug development and in immunological studies in human and experimental filariasis.

## Background

Lymphatic filariasis causes debilitating chronic hydrocele and/or lymphoedema in about 40 million people world wide – nearly 120 million people are found infected with the nematodes, about 90% with *W.bancrofti *and the rest with *B.malayi*, mostly in tropical countries. Infective larvae (L3) from mosquitoes enter the mammalian host and develop into male and female adult stage parasites in the lymphatics. After mating the adult female worms release thousands of microfilariae (Mf) that enter the blood circulation for further development in mosquitoes. In the absence of intermediate animal hosts, the process of embryogenesis leading to fecundity of adult female worms is very critical for persistence of these obligate parasites in human communities.

Morphologically, different intrauterine developmental stages are discernable in the uterine cavity of adult female worms. Eggs or oocytes after fertilization with sperms transform into motile microfilariae and are released by the adult female worms [1]. Currently, tools are not available to quantify the different developmental stages of embryogenesis other than approximate scoring by microscopy [2,3]. Development of precise assays for monitoring embryogenesis in adult female worms have the potential to address crucial issues in filariasis research – filarial worms are known to harbour endosymbionts such as *Wolbachia*, which play a significant role in fecundity of adult filarial worms [3,4]. Tetracycline or doxycycline treatment of the infected hosts effectively eliminates the endosymbionts resulting in inhibition of embryogenesis in female worms [5]. Inhibition of embryogenesis in infected human hosts can be scored only by monitoring decrease/loss of peripheral microfilaraemia-lymphatic dwelling adult stage parasites are not accessible for study. However in experimental animal models the adult female worms can be harvested and dissected *in vitro *and the intrauterine stages can be approximately scored by microscopy [2,3]. In this communication we describe a flow cytometry based method for studying embryogenesis in adult female filarial worms. The utility of this method for quantifying binding of lectins and antibodies to different intra uterine stages of filarial parasites has also been evaluated.

## Methods

### Preparation of intra-uterine stages for flow cytometry

Adult female filarial worms, *Setaria digitata *were collected from the peritoneum of cattle at a nearby abattoir in sterile alpha – MEM containing 1% glucose, Penicillin-100 units/ml, Streptomycin-100 μg/ml Gentamycin-50 μg/ml and Amphotericin-B 2.5 μg/ml and transported to the laboratory and used on the same day. Individual worms taken in a petridish were washed three times (x3) in sterile medium and dissected into small pieces in about 5 ml of medium and incubated at 37°C for 30 mins. The large pieces were removed and the medium containing intra-uterine stages were harvested, washed in MEM and the final pellet of cells suspended in 1 ml of sheath fluid and analyzed using a flowcytometer (FACSCalibur, Becton Dickinson, USA) using Cell Quest Pro software. The data (5000 events) were acquired for forward and side scatter using the following settings: FSC, voltage E00 and SSC, voltage 340. The three populations were gated and sorted using a FACS sorter under moderate fidelity settings. The sorted suspensions were centrifuged on to microscopic slides using a cytospin and observed under a phase contrast microscope for identification of organisms in the gated populations.

### Collection of blood for sera from human filariasis cases

Blood for sera were collected from patients with chronic filariasis (elephantiasis/hydrocoele) and microfilariae carriers from filariasis endemic areas near Bhubaneswar as described by us earlier [6]. Sera stored at -20°C were diluted in PBS with 1% BSA and used for binding to intra-uterine stages as described above.

### Preparation of immune sera against intrauterine stages

Five *Mastomys coucha *were immunized with three doses (15 days apart) of intra-uterine stages (50,000 cells per dose) in complete Freund's adjuvant and blood for sera was collected between days 40–45 and tested for antibody reactivity to intrauterine stages as described above.

### Purification of circulating microfilariae from infected blood

Microfilariae (Mf) from human blood were purified using 5 μM pore polycarbonate membranes as described by us earlier [6]. Nocturnal blood samples collected from *W.bancrofti *infected Mf carriers or day time blood samples from *S.digitata *infected cattle were used for purification – Mf washed in Tris-EDTA buffer were taken in sheath fluid and analyzed by flow cytometry for forward and side scatter as described above.

### Lectin binding assay

A suspension (500 μl) containing about 10,000 intra uterine stages in PBS 7.2 with 1% BSA were mixed with 500 μl of 4 μg/ml or 1 μg/ml of biotinylated WGA (L-5142, Sigma Chemical Co, USA). Simlarly two dilutions, 500 or 2000 fold diluted biotinylated Con-A (BA-16, Bangalore Genie, India) were used and the suspensions were incubated at RT for 45 min. The cells were then washed x3 in PBS and taken in 0.5 ml of PBS with 1% BSA to which 0.5 ml of 250 fold diluted Avidin – FITC (S-3762, Sigma Chemical Co, USA) was added and incubated for 30 min at RT. The suspension was washed again thrice in PBS and samples taken in 1 ml of sheath fluid and 5000 events were acquired. The three populations were gated and fluorescence intensity was read using a 488 nm laser. The percentage reactivity of lectins in comparison to Avidin-FITC controls was calculated using CellQuest Pro software.

### Antibody binding assay

A suspension (500 μl) containing about 10,000 IU stages in PBS-BSA were mixed with 500 μl of 50 fold diluted human or *Mastomys *sera (normal as well as immunized animals) and incubated at RT for 45 min. The cells were then washed x3 and taken in 0.5 ml of PBS-BSA to which 0.5 ml of 250 fold diluted FITC labeled anti-human Immunoglobulin (F-6506, Sigma Chemical Co.USA) or 50 fold diluted FITC labeled anti-mouse IgG was added and incubated for 30 min at RT. The suspension was washed x3 in PBS and organisms were taken in 1 ml of sheath fluid for acquiring 5000 events in FACS Calibur. The three populations were gated and fluorescence intensity was read using a 488 nm laser. The percentage reactivity as well as mean fluorescence intensity were calculated for antibody binding and compared with FITC labeled second antibody conjugate controls using CellQuest Pro software.

## Results

### Forward and Side scatter and identity of stages

**Figure 1:**
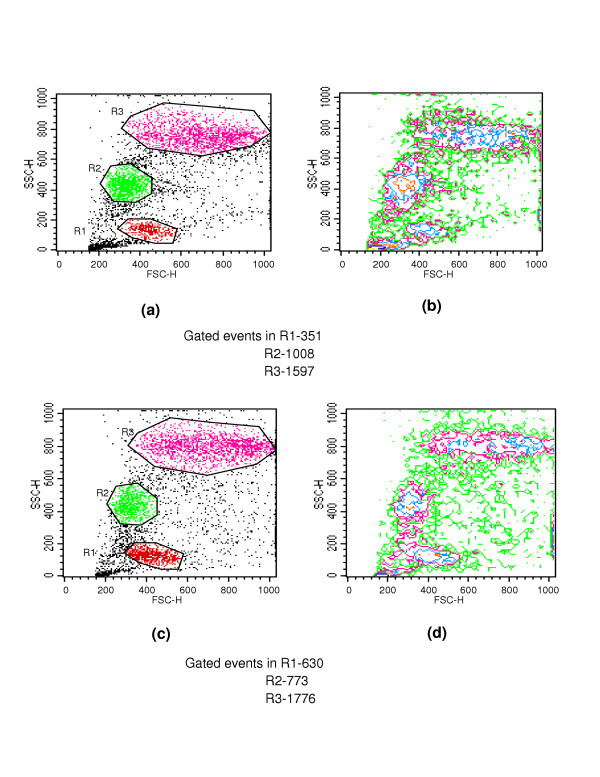
Three populations R1, R2 and R3 in forward (FSC, voltage E00) and side scatter (SSC, voltage 340) of intra-uterine stages; two representative worms by Flow cytometry are shown: **1a and 1c **– Dot plots; **1b and 1d **– respective contour maps; the number of events in individual gates for IU stages are indicated below respective figures.

**Figure 2:**
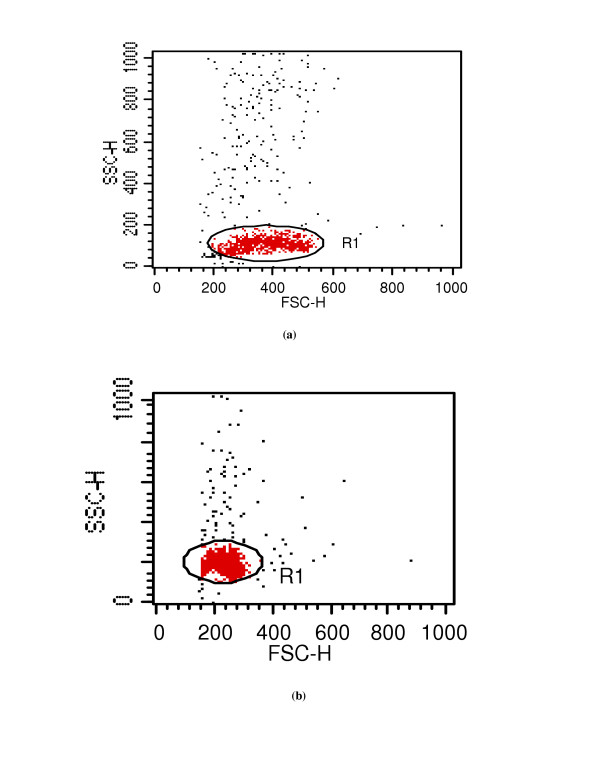
Dot plots for purified Mf of *S.digitata *(**2a**) and *W.bancrofti *(**2b**) in forward and side scatter.

**Figure 8:**
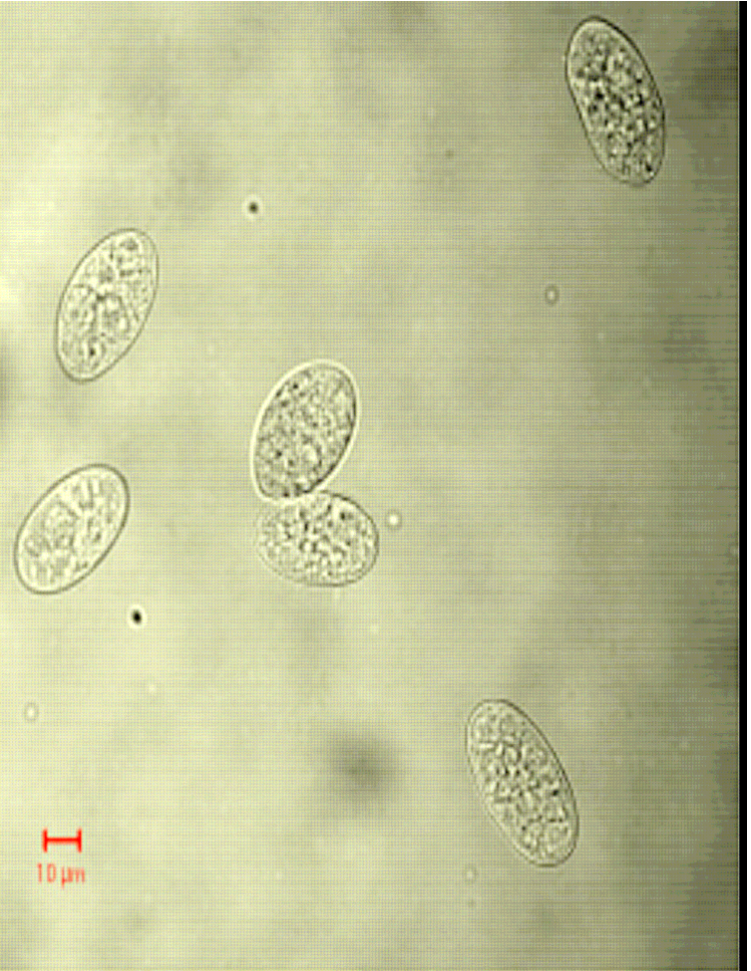
Sorted R2 population showing early developmental stages of eggs.

**Figure 9:**
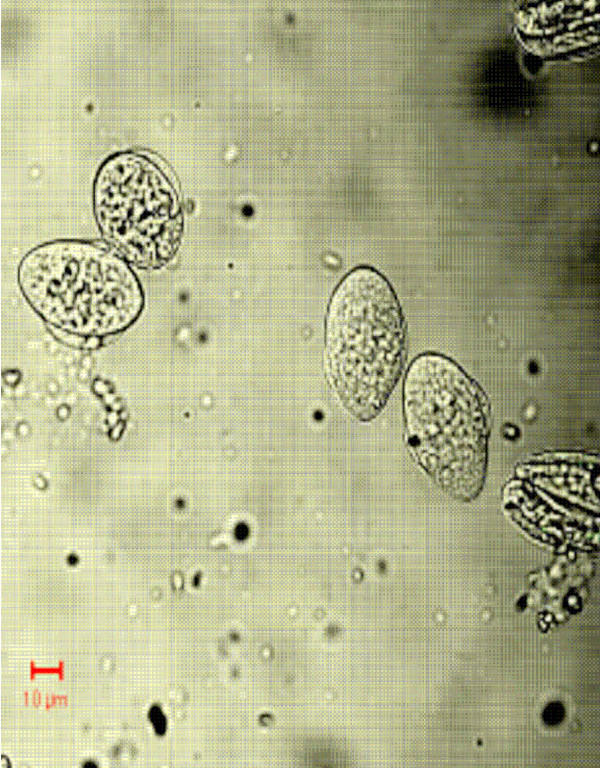
Sorted R3 cells of late developmental stages of eggs.

**Figure 10:**
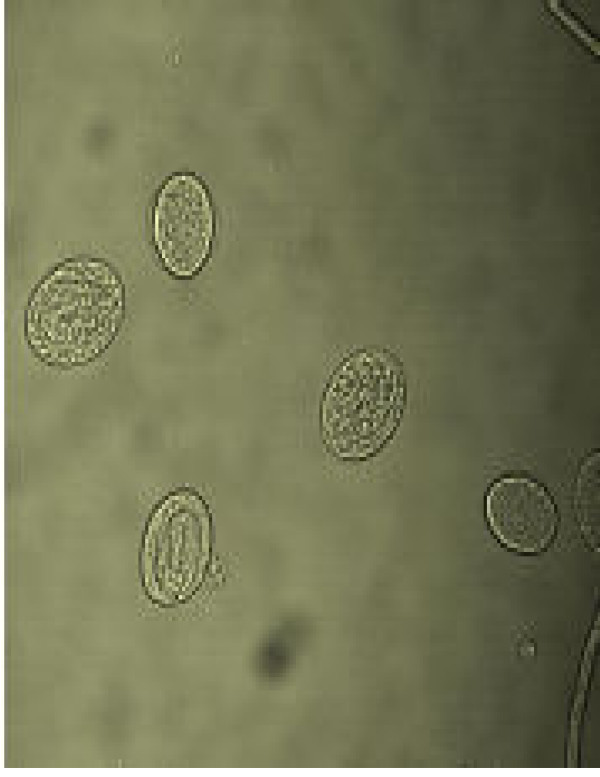
Phase contrast microscopic image of unsorted population of intrauterine stages.

**Figure 11:**
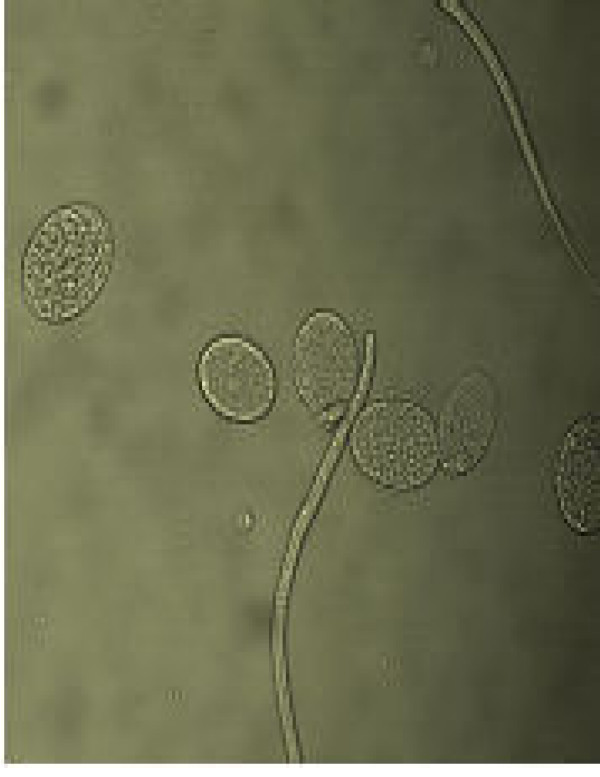
Phase contrast microscopic image of unsorted population of intrauterine stages.

**Figure 12:**
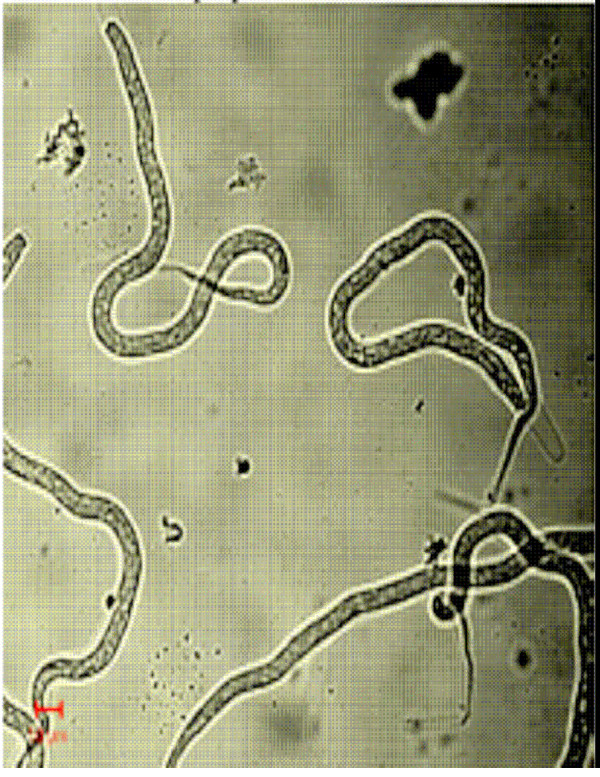
Sorted R1 cells showing pure Mf.

**Figure 13:**
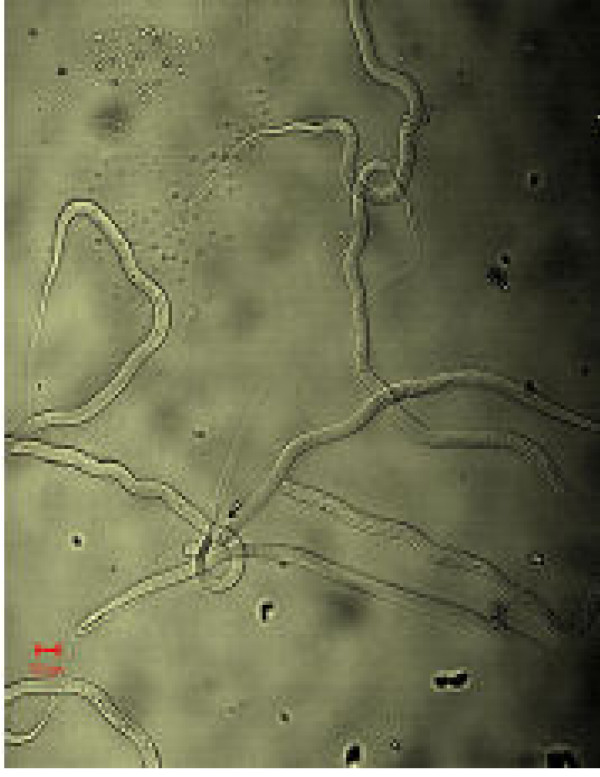
Sorted R1 cells showing pure Mf.

Figs 1a &1c show the dot plots of intrauterine stages in forward and side scatter for two representative adult worms; respective contour maps are shown in Fig 1b &1d. Three distinct populations of organisms, R1, R2, and R3 could be identified. The three populations were consistently found in several worms although the relative numbers in each scatter varied between worms as shown in Fig 1. Purified mf of *S.digitata *and *W.bancrofti *(from blood) by using nucleopore membrane filtration scattered exclusively in the R1 region indicating that organisms in this region are microfilariae (Fig 2a and 2b respectively). The organisms in the three scatter groups were purified by sorting them in a FACS Calibur sorter and examined under a phase contrast microscope: Figs 10 &11 show organisms in the unsorted population which contain a mixture of early and late developmental stages of eggs as well as fully stretched Mf, while Fig 12 and 13 reveal pure Mf of *S.digitata *in the sorted R1 population confirming the data presented in fig 2a and 2b above. Organisms in R2 gate were found to be early developmental stages of eggs (Fig 8) while late developmental stages were found in the R3 population (Fig 9).

### Lectin binding to intra-uterine stages

**Figure 3:**
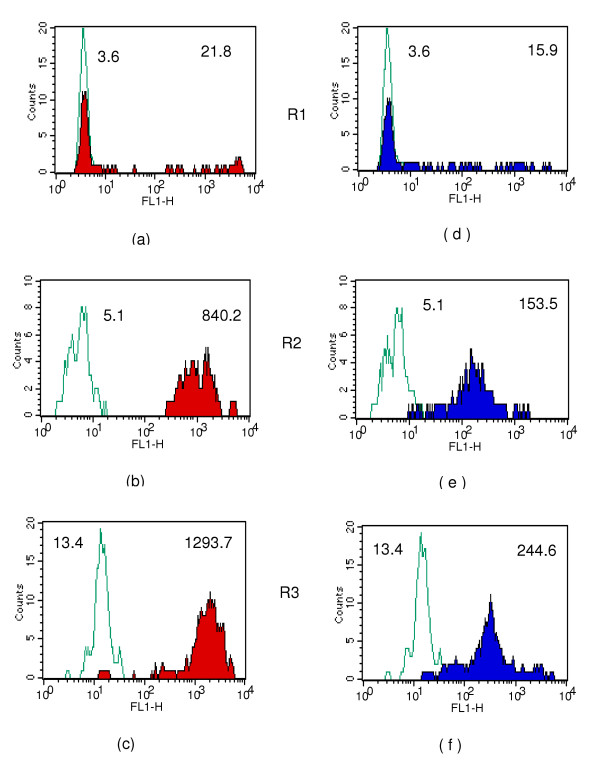
Con-A binding to intra-uterine stages: single colour analysis using 488 nm laser; **3 a,b and c: **Con-A (500 fold diluted) reactivity to R1,R2 and R3 gated populations. **3 d, e and f: **Con-A (2000 fold diluted) reactivity to R1, R2 and R3 gated populations. Green line: Avidin-FITC control; coloured shaded areas: specific reactivity of Con-A. Numbers shown on top left and top right on histograms represent geometric mean intensity of fluorescence for control and Con-A respectively.

**Figure 7:**
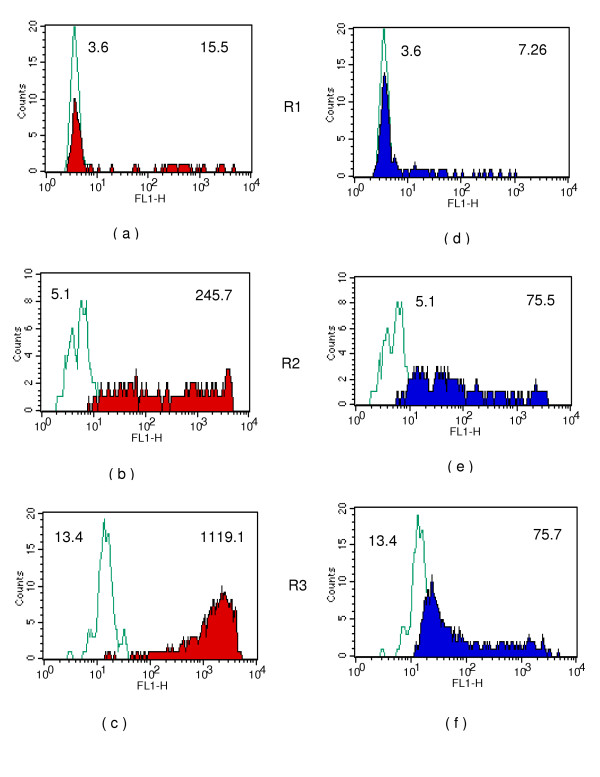
Binding of WGA to intra-uterine stages: single parameter analysis using 488 nm laser; **7 a,b and c: **WGA (2 μg/ml) reactivity to R1,R2 and R3 gated populations. **7 d,e and f: **WGA (0.5 μg/ml) reactivity to R1,R2 and R3 gated populations. Green line: Avidin-FITC control; coloured shaded areas show specific reactivity of WGA. Numbers shown on top left and top right on histograms represent geometric mean intensity of fluorescence for control and WGA respectively

Two lectins, Wheat Germ Agglutinin (WGA) and Conconavalin-A (Con-A) that specifically react primarily with N-Acetyl-D-Glucosamine and D-mannose residues respectively (which are present ubiquitously in several parasites including filarial parasites), were chosen as markers in this study to evaluate the flow cytometry based assay procedure. The intrauterine stages incubated with biotinylated WGA or Con-A were analysed for single colour fluorescence using 488 nm laser in the three gated population. The background reactivity and mean fluorescence reactivity of avidin-FITC (controls) for the three gated populations was minimal. The specific binding of WGA to the three populations is shown in Fig 7a–f. There was a dose dependant binding of WGA to intra-uterine stages – lower concentrations Fig 7d,e &7f) bound proportionately less than higher concentrations as shown in histograms in Fig (7a,b &7c). Similarly a dose dependent binding of Con-A to intrauterine stages could also be demonstrated (Fig 3a–f). Both the lectins bound significantly (> 95%) to early and late developmental stages of eggs (R2 and R3 respectively) – their reactivity to intra-uterine Mf (R1) was, however, not high.

### Antibody binding to Intra-uterine stages

**Figure 4:**
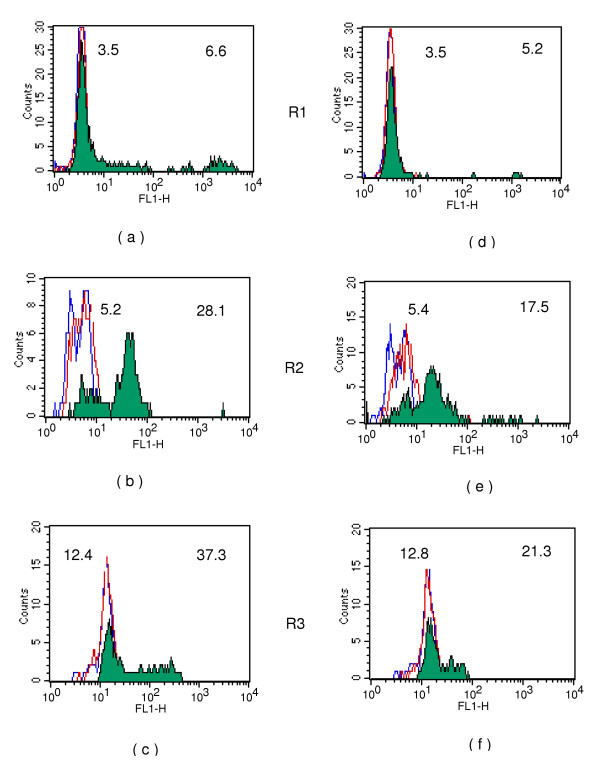
Antibodies binding in sera of *M.coucha *to three gated populations (R1,R2 and R3) of intra-uterine stages. **Fig 4 a,b and c **for one animal and **Fig 4 d,e and f **for a second animal are shown in the histogram. Blue lines: Anti-mouse IgG-FITC conjugate control; red lines: Pre-immune sera; Coloured shaded areas: antibody reactivity in respectiveimmunized sera. Numbers shown on top left and top right on histograms represent geometric mean intensity of fluorescence for pre and post-immunized sera respectively.

**Figure 5:**
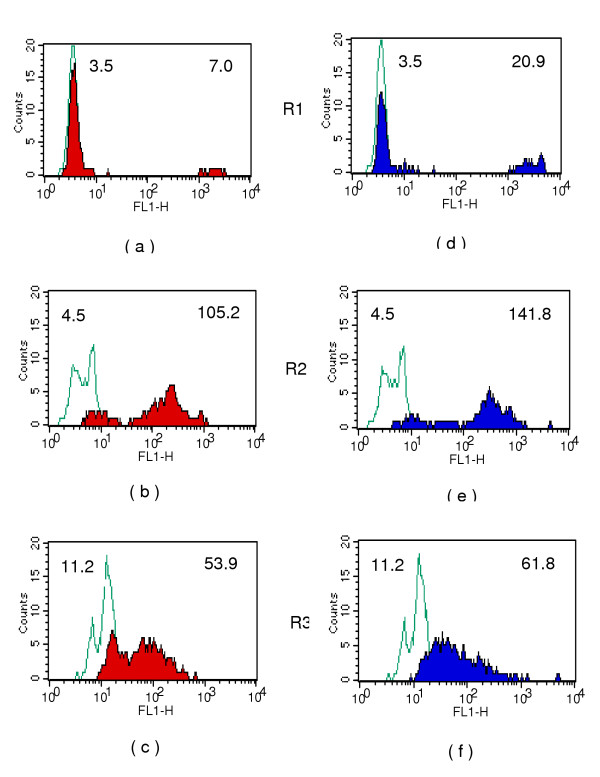
Antibody binding in two patients with chronic filariasis: Histograms showing reactivity to the three gated populations (R1,R2 and R3) of intra-uterine stages. **Fig 5 a,b and c **for one human serum and **Fig 5 d,e and f **for another patient. Blue line: Anti-human IgG-FITC control; Colour shaded areas: Antibody reactivity in test sera; Numbers shown on top left and top right on histograms represent geometric mean intensity of fluorescence for control and test sample respectively.

The binding of antibodies in human Bancroftian filariasis and in *S.digitata *immune *Mastomys *sera to intrauterine developmental stages could also be studied. The results of single colour fluorescence using 488 nm laser in the three-gated populations in two *Mastomys *sera are shown in Fig 4. The background binding of anti-mouse IgG-FITC conjugate to the three gated populations R1, R2, and R3 was very minimal. The binding profile of pre-immune sera were similar to conjugate controls while significant binding of antibodies to intrauterine eggs (R2 and R3 populations) could be demonstrated in both the immune sera (Fig 4a–f), in addition, similar reactivity was shown in three other immune *Mastomys *sera also (data not shown). Binding of antibodies in human filariasis sera to intrauterine stages could also be shown by the assay. The assay for two sera of elephantiasis cases is shown in Fig 5a–f. Significant binding of antibodies to intrauterine stages, particularly to egg stages could be demonstrated.

**Figure 6:**
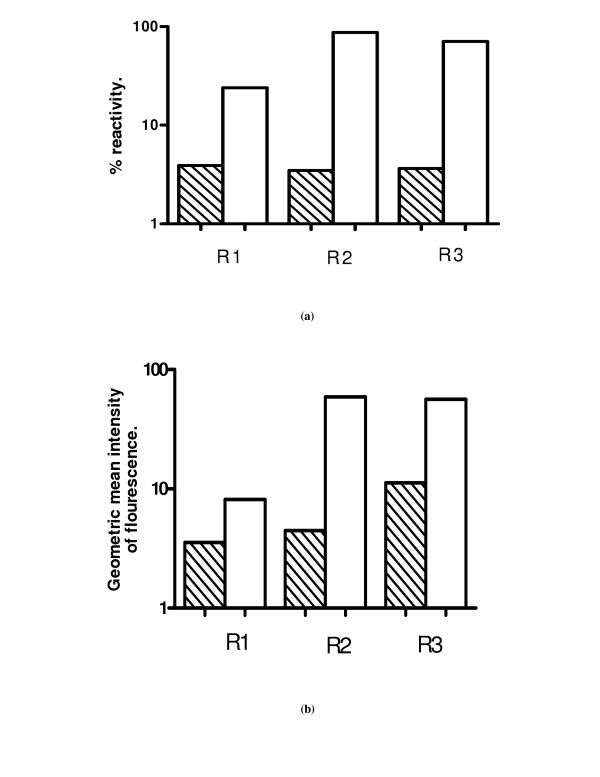
Antibody binding in chronic filariasis cases; **Fig 6 (a): **Mean percentage reactivity of antibodies in nine subjects to different intra-uterine stages. **Fig 6 (b): **Mean Geometric fluorescent intensity for the same sera depicted in **6a**. Striped bars: Anti-human IgG-FITC conjugate control; Open bars: test samples.

Although antibodies in human sera did not bind well to intra-uterine Mf, very significant binding of antibodies to a small select population of Mf was demonstrable by the assay (Fig 5a &5d). The mean antibody levels in nine patients with chronic filariasis were quantified (Fig 6). The levels were expressed as percentage reactivity and mean geometric fluorescence intensity as shown in Fig 6a &6b respectively. The antibody and lectin binding reported in this study are primarily restricted to reactivity of these molecules to the surface of intra-uterine stages since fresh live stages collected from adult female worms were used for the assays. Fixation with formaldehyde and permeabilising the cells resulted in antibody binding to intracellular components also (data not shown).

## Discussion

Embryogenesis is central to the biology of helminths and could be an effective target for devising intervention strategies for blocking transmission of parasitic helminths from mammalian hosts. Immunological studies in human as well as experimental filariasis have been largely directed towards host responses against infective larvae, microfilariae and adult stage parasites [7,8]. However, very few studies have addressed antigens expressed in/on intrauterine developing stage [9]. We consider the non-availability of sensitive assays to undertake studies on antibody responses to intrauterine stages as one of the reasons for such a limited number of studies. The large size of intrauterine stages (about 50 μM for egg stages and about 160 μM for Mf) do not limit the applicability of this novel assay since no special modification in the flowcytometer is required, the dimensions of standard flowcell provided by the manufacturers in FACSCalibur model was sufficient to perform the assays. We envisage a wide application for this new flowcytometry-based method of monitoring embryogenesis as well as antibody binding to intra-uterine stages in filariasis research: a) removal of endosymbionts in human hosts by doxycycline requires administration of the drug over a period of several weeks (5) and there is a need for developing newer anti-rickettsial drugs for blocking embryogenesis/fecundity in adult filarial worms; the new method described in this communication can be effectively used for screening a large number of potential compounds/drugs; b) although doxycycline and tetracycline have been demonstrated to eliminate endosymbionts in adult filarial worms, the precise intrauterine stage at which embryogenesis is blocked is not known and can now be studied with this new assay; c) cytokines such as IL-4 and molecules of innate immunity such as TLR-4 have been demonstrated to play a role in regulating production of microfilaraemia in experimental hosts [4,10]. Adult filarial worms can now be implanted in mice made deficient for specific cytokine gene expression or transgenic for such host molecules to monitor their role in embryogenesis; d) several filarial antigens have been cloned, sequenced and expressed in recent years and some have been used as putative vaccine candidates in experimental models [11]; e) high reactivity of antibodies in human filariasis sera to a select population of Mf (Fig 5a &5b) could be due to polymorphic antigens expressed on Mf sheath described by us several years ago (12). The novel procedure described here could assist in selectively sorting such reactive population of Mf for genetic analysis of polymorphic filarial antigens. The effect of induction of immune response to such recombinant molecules on embryogenesis can be studied using the assay described in this communication; e) filariasis in human communities presents with a variety of clinical and parasitological features. Infection is characterized by presence of circulating Mf and/or antigenemia and diseased subjects display acute and/or chronic clinical manifestations. Studies on immune responses of these patients to different developmental stages viz., infective larvae, adult stage parasite and Mf have indicated significant differences between the groups [summarized in [7]]. The new assay reported in this communication can be expected to now allow quantification of antibody responses to different intra-uterine stages of filarial parasites in human filariasis. The principles of flow cytometry have been recently used for separation and monitoring death/survival of another nematode *C.elegans *[13]. It may now be possible to study programmed cell death of developing intra-uterine stages in filarial parasites.

A bovine filarial parasite *S.digitata *has been used for establishment of the new assay system in this study. The assay could also be used for microfilariae of *W.bancrofti *(purified from infected endemic subjects). It is however, essential to evaluate the utility of this flowcytometry based method for other more commonly used filarial parasites, viz., *Brugia malayi, Brugia pahangi, Dirofilaria immitis, Litomosoides sigmodontis *etc.

## Conclusion

The manuscript reports a novel flow cytometry based method to monitor progression of embryogenesis in adult filarial worms. Apart from relative quantification of different intra uterine developmental stages, the assay allows quantitative binding of lectins and antibodies to each of the intrauterine stages. It may now be possible to monitor levels of antibodies in infected as well as immune hosts to intra-uterine developmental stages that could become a parameter for monitoring anti-fecundity immunity in filariasis, the assay can therefore be used as a powerful tool for drug development and in immunological studies in human and experimental filariasis.

## Competing interests

The author(s) declare that they have no competing interests.

## Authors' contributions

BRS: Conducted significant part of the experimental work including the assays using flowcytometer, compiled the data and wrote the draft manuscript.

ADM: Conducted part of the experimental work including the assays using flowcytometer, performed the immunoassays.

AM: Purified the stages using the sorter of flowcytometer, performed cytospin and captured phase contrast images for the sorted population.

PKD: Participated in experiments involving the use the flowcytometer sorter and use of confocal/ phase contrast microscopy

BR: Conceived the idea, designed experiments, interpreted the data and completed the manuscript
